# Genotypic antimicrobial resistance characterization of *E. coli* from dairy calves at high risk of respiratory disease administered enrofloxacin or tulathromycin

**DOI:** 10.1038/s41598-020-76232-w

**Published:** 2020-11-09

**Authors:** R. V. Pereira, C. Foditsch, J. D. Siler, S. C. Dulièpre, C. Altier, A. Garzon, L. D. Warnick

**Affiliations:** 1 Department of Population Health and Reproduction, College of Veterinary Medicine, University of California Davis, Davis, CA 95616 USA; 2 Department of Population Medicine and Diagnostic Sciences, College of Veterinary Medicine, Cornell University, Ithaca, NY 14850 USA

**Keywords:** Antimicrobial resistance, Preventive medicine, Molecular biology

## Abstract

The objective of this study was to evaluate the longitudinal effect of enrofloxacin or tulathromycin use in calves at high risk of bovine respiratory disease (BRD) on antimicrobial resistance genes and mutation in quinolone resistance-determining regions (QRDR) in fecal *E. coli*. Calves at high risk of developing BRD were randomly enrolled in one of three groups receiving: (1) enrofloxacin (ENR; n = 22); (2) tulathromycin (TUL; n = 24); or (3) no treatment (CTL; n = 21). Fecal samples were collected at enrollment and at 7, 28, and 56 days after beginning treatment, cultured for *Escherichia* *coli* (EC) and DNA extracted. Isolates were screened for cephalosporin, quinolone and tetracycline resistance genes using PCR. QRDR screening was conducted using Sanger sequencing. The only resistance genes detected were *aac(6′)Ib-cr* (n = 13), *bla*-CTX-M (n = 51), *bla*-TEM (n = 117), *tet*A (n = 142) and *tet*B (n = 101). A significantly higher detection of *gyr*A mutated at position 248 at time points 7 (OR = 11.5; *P* value = 0.03) and 28 (OR = 9.0; *P* value = 0.05) was observed in the ENR group when compared to calves in the control group. Our findings support a better understanding of the potential impacts from the use of enrofloxacin in calves on the selection and persistence of resistance.

## Introduction

When quinolone drugs were introduced, a delay in the development of resistance was expected because as synthetic antimicrobial agents, bacteria would not be exposed to quinolone resistance genes occurring in natural environments. The first quinolone drug used in human medicine was nalidixic acid in 1965, while the first fluoroquinolone drug approved for use in veterinary was enrofloxacin in 1989^1^. Moreover, it was predicted that the only mechanism for achieving clinically significant resistance would be rare simultaneous mutations in two or more target genes^2^. Nevertheless, growing concerns with the emergence and spread of Enterobacteriaceae showing reduced susceptibility to quinolone drugs support the need to understand how the use of fluoroquinolones as antimicrobials influence the process of selection and emergence of resistance^3^.

A recent study has shown that a single label dose of enrofloxacin in preweaned calves with the aim of reducing the risk of bovine respiratory disease could result in a significantly higher shedding of fecal *E. coli* resistant to ciprofloxacin when compared to calves not being treated with this drug^4^. Increase resistance to quinolone drugs is of high relevance to human medicine, because it is a critically important antimicrobial, and is commonly used for the treatment of many serious infections in humans, including severe cases on the salmonellosis, a potential food-borne pathogen^5^. Given the current unknows for the potential role that cattle may have on the selection of quinolone resistance enteric bacteria, more research is needed to identify specific mechanism of resistance that may be selected at a higher incidence due to the use of fluoroquinolone drugs; this information is important to generate evidence-based data to evaluate impacts of using fluoroquinolone drugs in cattle, as well as propose targeted interventions if needed.

A study by Gomez-Gomez et al. (1997) provided laboratory evidence that plasmid-mediated quinolone resistance (PMQR) was possible in *E. coli* despite the mechanism not being found in nature^6^. Using multidrug resistance (MDR) systems active against quinolones, Martinez et al. (1998) demonstrated in vitro how quinolone-resistant bacteria and transferrable quinolone resistance genes could exist in the environment and serve as a reservoir^7^. The first PMQR gene, *qnr*, was found on a multi-resistance plasmid in a clinical isolate of *Klebsiella pneumoniae*^8^. Five families of *qnr* that encode DNA gyrase protection proteins have been described in plasmids from bacterial pathogens: *qnrA*, *qnrB*, *qnrS*, *qnrC,* and *qnrD.* Other PMQR genes are efflux pumps, *oqxAB* and *qepA*, and a variant of an aminoglycoside acetyl transferase*, aac(6′)-Ib-cr*^2,9–11^.

PMQR genes have been shown to be associated with the similar mobile genetic elements as those of extended spectrum β-lactamase (ESBL) in *E. coli* causing urinary tract infection^12^. Use of quinolone drugs as a selection pressure for selection of quinolone resistance bacteria in clinical setting has been reported by many researchers, including the potential for co-selection to multidrug resistant isolates associated with quinolone resistance^13,14^.

The objective of this study was to evaluate the longitudinal effect of enrofloxacin or tulathromycin use in preweaned calves at high risk of bovine respiratory disease on the prevalence of antimicrobial resistance genes and mutation in QRDR in fecal *E. coli*. We hypothesized that the treatment of calves with enrofloxacin would significantly increase the prevalence of fecal *E. coli* carrying genomic elements conferring resistance to fluoroquinolones when compared to isolates from calves treated with drugs belonging to another common drug class used in this scenario (tulathromycin) or to a control group.

## Results

A total of 84 animals were enrolled in the study. From these, 18 calves were excluded due to treatment for diarrhea or loss to follow up, with 6 being from the ENR group (n = 22), 7 from the CTL group (n = 21), and 5 from the TUL group (n = 24). A total of 67 calves from the initial 83 remained in the study. Only 17 of the 108 QRDR mutations detected in the study were identified in isolates with phenotypic resistance to quinolone drugs (Supplemental Table 3). The distribution for the accumulated QRDR mutations observed in individual isolates (QRDR profiles) is outlined in supplemental Table 4.

The most common mutation detected for both isolates which did or did not display phenotypic resistance to ciprofloxacin, was *gyr*A 594 (point mutation T → C). Five QRDR mutations presented a significantly higher risk for being identified in ciprofloxacin resistant isolates: *par*C 239 (G → T), *gyr*A 248 (C → T), *gyr*A 259 (G → A), *gyr*A 570 (C → T), and *par*E 1372 (T → G). No significant effect was observed for the association between QRDR profiles risk due to treatment group by time point. Furthermore, *E. coli* isolates with an *aac(6′)Ib-cr* gene had the same following simultaneous mutations: *parC* 239 (G → T), *gyrA* 248 (C → T) and *gyrA* 259 (G → A).

**Figure 1:**
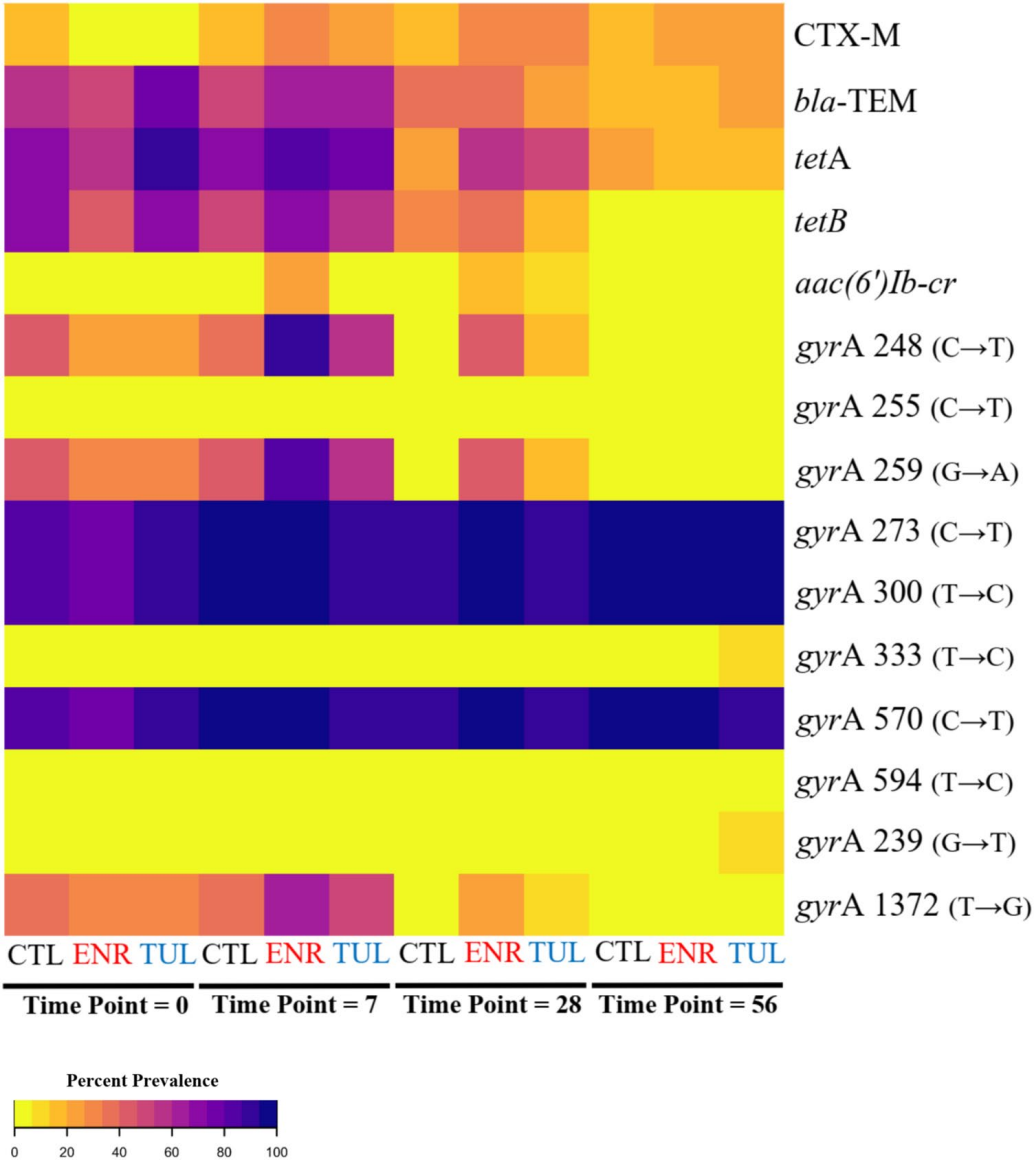
Heatmap of percent distribution by treatment group and time point for prevalence of antimicrobial resistance gene and QRDR mutations that were present in at least one isolate with phenotypic resistance to ciprofloxacin. Created using RStudio version 1.2.5033 (https://rstudio.com/).

A heatmap with the distribution of antimicrobial resistance gene and QRDR mutations detected in isolates with phenotypic resistance to ciprofloxacin is displayed in Fig. 1. From the 264 *E. coli* isolates in the study, the only PMQR gene detected in isolates in the study was *aac(6′)Ib-cr*. From the thirteen isolates detected with this gene, only one was in the control group at time point 28 (1/21), nine were observed in the ENR group at time points 7 (n = 5/22) and 28 (n = 4/22), and three were in the TUL group at time points 7 (n = 1/24) and 28 (n = 2/24) (Table 1). All thirteen isolates carrying this PMQR gene were observed to also have these three point mutations: *par*C 239 (chromosomal position) (point mutation G → T), *gyr*A 248 (C → T), and *gyr*A 259 (G → A). Fisher exact test revealed that calves in the ENR group (n = 5/22) had a significantly higher number of *aac(6′)Ib-cr* at time point 7 when compared to the CTL group (n = 0/21) (*P* value = 0.048). The only other resistance gene with a statistical significance for the Fisher exact test was for the ENR group which had a significantly higher number of *tet*A genes (n = 13/22) at time point 28 when compared to the CTL group (n = 5/21) (*P* value = 0.03).

**Table 1:** Sensitivity and specificity of genotype predictions of resistant antimicrobial phenotype for *E. coli* isolates by time point and treatment group for antimicrobial resistance genes *aac(6′)Ib-cr*(A). 1. Time point in days when sample was collected; 2. Treatment group; 3. Number of isolates from each treatment group for each sampling time point; 4. Number of isolates for which both the referred antimicrobial resistance gene and corresponding resistance phenotype were observed; 5. Number of isolates for which the referred antimicrobial resistance gene was not detected, but the expected corresponding resistance phenotype were observed; 6. Number of isolates for which the referred antimicrobial resistance gene was detected, but the expected corresponding resistance phenotype was not observed; 7. Number of isolates for which both the referred antimicrobial resistance gene and corresponding resistance phenotype were not observed; 8. Sensitivity for the referred resistance gene to correctly predict phenotypic resistance to the corresponding antimicrobial drug; 9. Specificity for the absence of the referred resistance gene and prediction of lack of phenotypic resistance to the corresponding antimicrobial drug.

			*aac(6′)Ib-cr*					
Time point^1^	Tx group^2^	N° isolates^3^	G+: P+^4^	G−: P+^5^	G+: P-^6^	G−: P−^7^	Se^8^	Sp^9^
0	CTL	21	0	9	0	12	0.0	100
	ENR	22	0	6	0	16	0.0	100
	TUL	24	0	7	0	17	0.0	100
7	CTL	21	0	9	0	12	0.0	100
	ENR	22	5	14	0	3	26.3	100
	TUL	24	1	13	0	10	7.1	100
28	CTL	21	1	0	0	20	100.0	100
	ENR	22	3	6	1	12	33.3	92.3
	TUL	24	2	2	0	20	50.0	100
56	CTL	20	0	0	0	20	–	100
	ENR	20	0	0	0	20	–	100
	TUL	23	0	0	0	23	–	100

For the genes detected in isolates in the study, tables were used to display sensitivity and specificity of genotype predictions of resistant antimicrobial phenotype for *E. coli* isolates by time point and treatment group for antimicrobial resistance genes *aac(6′)Ib-cr *(Table 1), CTX-M and *bla*-TEM (Table 2), and *tet*A and *tet*B (Table 3). The antimicrobial resistance gene *bla*OXA was not detected in the isolates tested.

**Table 2:** Sensitivity and specificity of genotype predictions of resistant antimicrobial phenotype for *E. coli* isolates by time point and treatment group for *CTX-M and bla-TEM* genes. 1. Time point in days when sample was collected; 2. Treatment group; 3. Number of isolates from each treatment group for each sampling time point; 4. Number of isolates for which both the referred antimicrobial resistance gene and corresponding resistance phenotype were observed; 5. Number of isolates for which the referred antimicrobial resistance gene was not detected, but the expected corresponding resistance phenotype were observed; 6. Number of isolates for which the referred antimicrobial resistance gene was detected, but the expected corresponding resistance phenotype was not observed; 7. Number of isolates for which both the referred antimicrobial resistance gene and corresponding resistance phenotype were not observed; 8. Sensitivity for the referred resistance gene to correctly predict phenotypic resistance to the corresponding antimicrobial drug; 9. Specificity for the absence of the referred resistance gene and prediction of lack of phenotypic resistance to the corresponding antimicrobial drug.

Time point^1^	Tx group^2^	N° isolates^3^	CTX-M						*bla*-TEM					
			G+: P+^4^	G−: P+^5^	G+: P−^6^	G−: P−^7^	Se^8^	Sp^9^	G+: P+^4^	G−: P+^5^	G+: P−^6^	G−: P−^7^	Se^8^	Sp^9^
0	CTL	21	3	13	0	5	18.8	100	21	0	0	0	100	−
	ENR	22	1	20	0	1	4.8	100	22	0	0	0	100	−
	TUL	24	0	22	0	2	0.0	100	24	0	0	0	100	−
7	CTL	21	4	13	0	4	23.5	100	21	0	0	0	100	−
	ENR	22	6	9	0	7	40.0	100	21	0	1	0	100	0.0
	TUL	24	5	15	0	4	25.0	100	24	0	0	0	100	−
28	CTL	21	4	16	0	1	20.0	100	21	0	0	0	100	−
	ENR	22	7	10	0	5	41.2	100	21	0	1	0	100	0.0
	TUL	24	8	12	0	4	40.0	100	24	0	0	0	100	−
56	CTL	20	3	15	0	2	16.7	100	20	0	0	0	100	−
	ENR	20	5	14	0	1	26.3	100	19	0	1	0	100	0.0
	TUL	23	5	13	0	5	27.8	100	20	0	3	0	100	0.0

**Table 3:** Sensitivity and specificity of genotype predictions of resistant antimicrobial phenotype for *E. coli* isolates by time point and treatment group for *tet*A and *tet*B genes. 1. Time point in days when sample was collected; 2. Treatment group; 3. Number of isolates from each treatment group for each sampling time point; 4. Number of isolates for which both the referred antimicrobial resistance gene and corresponding resistance phenotype were observed; 5. Number of isolates for which the referred antimicrobial resistance gene was not detected, but the expected corresponding resistance phenotype were observed; 6. Number of isolates for which the referred antimicrobial resistance gene was detected, but the expected corresponding resistance phenotype was not observed; 7. Number of isolates for which both the referred antimicrobial resistance gene and corresponding resistance phenotype were not observed; 8. Sensitivity for the referred resistance gene to correctly predict phenotypic resistance to the corresponding antimicrobial drug; 9. Specificity for the absence of the referred resistance gene and prediction of lack of phenotypic resistance to the corresponding antimicrobial drug.

Time point^1^	Tx group^2^	N° isolates^3^	*tet*A						*tet*B					
			G+: P+^4^	G−: P+^5^	G+: P−^6^	G−: P−^7^	Se^8^	Sp^9^	G+: P+^4^	G−: P+^5^	G+: P−^6^	G−: P−^7^	Se^8^	Sp^9^
0	CTL	21	14	5	0	2	73.7	100	15	4	0	2	78.9	100
	ENR	22	11	4	1	6	73.3	85.7	10	5	0	7	66.7	100
	TUL	24	21	2	0	1	91.3	100	17	6	0	1	73.9	100
7	CTL	21	15	1	0	5	93.8	100	10	6	0	5	62.5	100
	ENR	22	19	2	0	1	90.5	100	16	5	0	1	76.2	100
	TUL	24	18	3	0	3	85.7	100	14	7	0	3	66.7	100
28	CTL	21	5	7	0	9	50.0	100	6	6	0	9	50.0	100
	ENR	22	12	2	1	8	57.1	100	8	6	0	8	57.1	100
	TUL	24	12	2	0	10	28.6	100	4	10	0	10	28.6	100
56	CTL	20	4	1	1	14	80.0	93.3	1	4	0	15	20.0	100
	ENR	20	2	0	2	16	100.0	88.9	0	2	0	18	0.0	100
	TUL	23	4	0	0	19	100.0	100	0	4	0	19	0.0	100

The distribution of *E. coli* antimicrobial resistance gene profile and antimicrobial resistance phenotype profile is displayed in Table 4. Isolates phenotypically resistant to three or more drug classes were labeled as multidrug resistant (MDR). The most common genotypic resistance profile was *bla*-TEM *tet*A *tet*B (n = 73/264), with the most common corresponding phenotypic profile being isolates resistant to amoxicillin/clavulanic acid, amoxicillin, enrofloxacin, cefoxitin, ceftriaxone, chloramphenicol, ciprofloxacin, nalidixic acid, sulfisoxazole, tetracycline and trimethoprim sulfamethoxazole (53% of isolates in this resistance genotype). This multidrug resistant phenotype was also the most common phenotype profile (92% of isolates in this resistance genotype) for the fourth most common resistance genotype profile, and the only with the gene *aac(6′)Ib-cr*. This resistance genotype profile was *aac(6′)Ib-cr* CTX-M *tet*A (n = 13/264).

**Table 4:** Distribution of 264 *E. coli* antimicrobial resistance gene profile and antimicrobial resistance phenotype profile. The in parenthesis “n” indicated the number of isolates with the identified genotype profile. 1. Isolate level resistance phenotypes profile: Au, amoxicillin/Clavulanic acid; Am, Amoxicillin; Enro, enrofloxacin; Fox, cefoxitin; Cip, ciprofloxacin; Cro, ceftriaxone; Cho, chloramphenicol; Na, nalidixic acid; Str, streptomycin; Te, tetracycline; Sxt, trimethoprim/sulfamethoxazole; Sul, sulfisoxazole; 2. Isolate level resistance genotypes for genes *aac(6′)Ib-cr*, CTX-M, *bla*-TEM, *tet*A and *tet*B; 3. Percent distribution of the referred antimicrobial resistance genotype profile within each antimicrobial resistance phenotype profile.

Antimicrobial resistance Phenotype^1^/genotype^2^	*bla*-TEM *tet*A *tet*B (n = 73)	*tet*A (n = 37)	CTX-M (n = 22)	*bla*-TEM (n = 13)	*aac(6′)Ib-cr* CTX-M *tet*A (n = 13)	*bla*-TEM *tet*A (n = 13)	*tet*B (n = 13)	CTX-M *bla*-TEM *tet*B (n = 6)	*bla*-TEM *tet*B (n = 4)	CTX-M *tet*A (n = 4)	CTX-M *tet*B (n = 3)	CTX-M *bla*-TEM (n = 2)	CTX-M *bla*-TEM *tet*A *tet*B (n = 1)	*tet*A *tet*B (n = 1)
	%^3^	%^3^	%^3^	%^3^	%^3^	%^3^	%^3^	%^3^	%^3^	%^3^	%^3^	%^3^	%^3^	%^3^
AuAmEnroFoxCroChoCipNaSulTeSxt	**53**	0	0	0	**92**	0	0	0	0	**50**	0	0	0	0
AuAmFoxCroSulTeSxt	**12**	**62**	0	**6**	0	**46**	**15**	0	0	0	0	0	0	0
AuAmEnroFoxChoCipNaSulTeSxt	**33**	0	0	0	0	0	0	0	0	0	0	0	0	**100**
AmCro	0	0	**100**	0	0	0	0	0	0	**25**	0	0	0	0
AuAmFoxCro	0	**3**	0	**76**	**8**	**15**	0	0	0	0	0	**50**	0	0
AuAmFoxCroSulTe	0	**5**	0	0	0	0	**77**	0	**50**	0	**100**	0	0	0
AuAmCroChoSulTeSxt	0	0	0	0	0	0	0	**67**	0	0	0	0	**100**	0
AuAmFoxCroTe	0	**5**	0	0	0	**8**	0	0	**25**	0	0	0	0	0
AuAmFoxCroChoSulTeSxt	**1**	**8**	0	0	0	0	0	0	0	0	0	0	0	0
AmCroTe	0	0	0	0	0	0	0	**33**	0	0	0	**50**	0	0
AuAmFoxCroSulSxt	0	0	0	**18**	0	0	0	0	0	0	0	0	0	0
AuAmFoxSulTe	0	**3**	0	0	0	0	0	0	**25**	0	0	0	0	0
AuAmSulTe	0	**3**	0	0	0	**8**	0	0	0	0	0	0	0	0
AuAmFoxCroChoSulTe	0	**5**	0	0	0	0	0	0	0	0	0	0	0	0
AuAmFoxChoSulTe	0	**5**	0	0	0	0	0	0	0	0	0	0	0	0
AmCroSulTe	0	0	0	0	0	0	0	0	0	**25**	0	0	0	0
AuAmFoxSulTeSxt	0	0	0	0	0	**8**	0	0	0	0	0	0	0	0
AmChoSulTe	0	0	0	0	0	**8**	0	0	0	0	0	0	0	0
NaSulTe	0	0	0	0	0	0	**8**	0	0	0	0	0	0	0
Te	0	0	0	0	0	**8**	0	0	0	0	0	0	0	0

From all resistance genes screened in the study, none was identified as significantly associated with the treatment groups in the study when analyzed by sampling time point. Five of the QRDR mutations were observed to be significantly associated with treatment groups when using an initial chi-square analysis, namely *gyr*A 248, *gyr*A 259, *par*C 348, *par*C 239, and *par*C 273. Following analysis of QRDR mutations using a mixed logistic regression revealed that only *gyr*A 248 resulted in a significant association between treatment groups by time points, with a tendency observed for *par*C 239 and *par*C 273. Sensitivity and specificity of QRDR mutations and corresponding phenotypic resistance to ciprofloxacin for *E. coli* isolates are displayed in Table 5. The QRDR mutation profile with the higher sensitivity and specificity had simultaneous point mutations for *parC* 239 (G → T), *gyrA* 248 (C → T), *gyrA* 259 (G → A), *gyrA* 570 (C → T), and *parE* 1372 (T → G).

**Table 5:** Sensitivity and specificity of QRDR mutations observed at least in 3 or more isolates and corresponding phenotypic resistance to ciprofloxacin for *E. coli* isolates. 1. Quinolone resistant-determining regions (QRDR) mutation. Chromosomal mutation gene and location; 2. Percent of isolates presenting the QRDR mutation profile from all isolate with phenotypic resistance to ciprofloxacin. The count of isolates with the QRDR mutation profile is in parenthesis; 3. Number of isolates with the referred QRDR mutation and phenotypic resistance to ciprofloxacin; 4. Number of isolates without the referred QRDR mutation and phenotypic resistance to ciprofloxacin; 5. Number of isolates with the referred QRDR mutation and without phenotypic resistance to ciprofloxacin; 6. Number of isolates without the referred QRDR mutation and without phenotypic resistance to ciprofloxacin; 7. Sensitivity for the referred QRDR mutation to correctly predict phenotypic antimicrobial resistance to ciprofloxacin; 8. Specificity for the absence of the referred QRDR mutation and prediction of lack of phenotypic resistance to ciprofloxacin.

QRDR mutation profile^1^	% (n) CIP R^2^	G + : P + ^3^	G−: P + ^4^	G + : P−^1^	G−: P−^6^	Se^7^	Sp^8^
*parC_239 gyrA_248 gyrA_259 gyrA_570 parE_1372 gyrA_468 gyrA_273 gyrA_255 gyrA_333 gyrA_300*	75.3 (58)	58	20	1	185	74.3	99.5
*parC_23 9gyrA_248 gyrA_259 gyrA_468 gyrA_273 gyrA_255 gyrA_333 gyrA_300*	18.2 (14)	14	64	1	185	17.9	99.5

From the mixed logistic regression models conducted, the only model that detected a significant difference was for QRDR mutation *gyrA* 248 (C → T), with significantly higher odds for detection of this mutation in the ENR group at time point 7 (OR: 11.59; 95% CI 1.2–109.6; *P* value: 0.03) and a trend for significance at time point 28 (OR: 9; 95% CI 0.9–85; *P* value: 0.05) when compared to the CTL group, as shown in Table 6. At time point 0, significantly lower odds for detection of mutation *gyrA* 248 (C → T) in ENR group was detected when compared to the control group (OR:0.1; 95% CI 0.02–0.54).

**Table 6:** Odds ratio from the logistic regression model for QRDR point mutation *gyr*A 248 for each treatment group by sampling time point. 1. Time point in days when sample was collected; 2. Treatment group; 3. Number of isolates from each treatment group for each sampling time point; 4. Percent of isolates within the referred treatment group ant time point with the QRDR mutation *gyr*A 248; 5. Standard error of the coefficient; 6. Odds ratio and lower and higher 95% confidence interval for association of mutation *gyr*A 248 with the treatment group by sampling time point, with the control group as the reference; 7. *P* value for the odds ratio.

Time point^1^	Tx group^2^	N° isolates^3^	%^4^	Coefficient	*SE*^5^	*OR (95% CI)*^6^	*P value*^7^
0	CTL	21	42.9			Ref	
	ENR	22	22.7	− 2.27	0.84	0.1 (0.02–0.54)	**0.008**
	TUL	24	25.0	− 1.28	0.76	0.27 (0.06–1.25)	0.09
7	CTL	21	38.1			Ref	
	ENR	22	90.9	2.40	1.13	11.59 (1.22–109.65)	**0.03**
	TUL	24	58.3	0.96	0.81	2.63 (0.52–13.12)	0.23
28	CTL	21	4.8			Ref	
	ENR	22	45.5	2.19	1.13	9.01 (0.95–85)	**0.055**
	TUL	24	16.7	1.31	1.18	3.7 (0.35–38.47)	0.27

## Discussion

From all PMQR screened in the study only *aac(6′)Ib-cr* was detected, with a significantly higher prevalence of this gene in the ENR group at time point 7 when compared to the control group (Table 1). At time point 28, *aac(6′)Ib-cr* could be detected in all three treatment groups, however by day 56 none of the treatment groups isolates had this gene. The *aac(6′)Ib-cr* is a variant of the *aac(6′)Ib* that encodes an aminoglycoside acetyltransferase that confers reduced susceptibility to ciprofloxacin by N-acetylation at the amino nitrogen on its piperazinyl substituent^15^. The *aac(6′)Ib-cr* gene was first reported in 2003 and has since been reported from multiple geographical locations and sources^15–17^. Specific conditions that result in the selection of PMQR have been shown to be affected by various factors, including *E. coli* genotypes (e.g. strains) and the selective environment, which together can also influence the selection of strains and PMQR genes with variable fitness costs^18^. *E. coli* carrying *aac(6′)Ib-cr* have been shown to have a significant increase in fitness cost, independent of the presence or not of QRDR chromosomal mutations^19^. In our study, results indicate that ENR treatment resulted in a competitive advantage for selection of harboring *aac(6′)Ib-cr* gene at time point 7, which however had a limited temporal effect with this gene not being detected at significantly higher numbers compared to the control group by day 28. This finding could be explained by the limited longitudinal advantage conferred by enrofloxacin, but also could be linked to an increase in the microbial competitive environment.

Microbiota characterization of fecal samples of calves in this study was presented in a previous publication and did not show a significant microbial disruption at the phylum level^20^. However, lower level taxonomic dysbiosis at the species and strain level could have occurred, potentially allowing for an environment that would favor prevalence of bacteria with higher fitness cost. Furthermore, the absence of the *aac(6′)Ib-cr* gene at day 56 for any treatment group indicates the limited longitudinal effect of a potential selective advantage conferred to this gene in *E. coli* for calves in both ENR and TUL groups. This was also observed for other resistance genes and QRDR mutations, with resistance genotypes that were observed at higher number in the few first sampling time points quickly decreasing to marginal levels by day 56, as can be observed in the heatmap in Fig. 1. A factor to consider in this study is that it was conducted with preweaned calves at the time of enrollment, but the last samples collected at time point 56 represent post-weaned calf samples, and changes in the microbiota during this period should be considered. As has been shown by previous studies, calves that were never exposed to antimicrobial drugs during the weaning period show an increase in shedding of *E. coli* in the feces at 2–3 weeks of age, with a gradual decrease to negligible number as the enteric microbiota matures and the calf approximates weaning age^21^.

All *E. coli* isolates harboring the *aac(6′)Ib-cr* gene presented the same genotypic profile for resistance genes, namely *aac(6′)Ib-cr,* CTX-M, and *tet*A, and which correlated with a multidrug phenotypic resistance profile for 11 antimicrobials belonging to multiple drug classes, including cephalosporins, quinolones, sulfas, tetracyclines and aminopenicillins (Table 4). Furthermore, these isolates also had simultaneous chromosomal mutations on the quinolone resistance-determining regions (QRDR) *parC* 239, *gyrA* 248 and *gyrA* 259. This corroborates the hypothesis that these isolates were an outcome of clonal expansion of the same *E. coli* strain in the study population at specific time points due to favorable conditions. QRDR mutations have been shown to result in clonal expansion of specific resistant strains through a multistep mutation selection process^22,23^. This has been observed to occur, and results in large-scale expansion of fluoroquinolone resistant *E. coli* clones, as described in one study where a multistep process of gene transfer and recombination’s resulted in the rise of multidrug resistant clonal group ST1193 reported to cause multiple cases of uropathogenic fluoroquinolone resistant infections in humans caused by *E. coli*^24^. In our study, isolates with chromosomal mutation to *gyrA* 570 and *parE* 1372 in addition to the aforementioned QRDR mutation profile (Table 5) had the highest reliability for correctly identifying isolates with corresponding phenotypic resistance to ciprofloxacin, with a sensitivity of 74.4% (Table 5). These two mutation resistance profiles for QRDR represent 84% of the identified profiles in *E. coli* isolates, and the similarities support the hypothesis that the strains carrying these two QRDR resistant profiles may be the output of multiple stepwise genomic adaptations that *E. coli* carrying these profiles have undergone to adapt to environmental selective pressures.

The only individual QRDR mutation that was significantly associated with treatment group by time point was *gyrA* 248 (C → T), which had significantly higher odds for occurring in ENR group at time points 7 and 28 when compared to the control group (Table 6). Resistance mutations to quinolone drugs usually occur first in *gyr*A in gram-negative bacteria, and following that mutation, additional mutations in *gyr*A or mutations in *gyr*B or *par*C can occur and will further decrease susceptibility augment resistance, although, alone these mutations are usually ineffective in a bacterial cell with wild-type *gyr*A, because the most-susceptible target sets the level of susceptibility^25^. PMQR is due to *qnr* proteins that protect the target enzyme from quinolone action, but these gene can only confer low-level resistance that can support the promotion of the selection of mutational high level resistance^26^. Mutation in *gyr*A 248, resulting in resistance to quinolone drugs has previously been reported in *E. coli,* and *Salmonella* isolates from both humans and animals^27,28^. One study screening 818 *E. coli* clinical isolates (77% resistant to nalidixic acid) from cows, chicken and pigs revealed that the most common nucleotide mutation site in *gyr*A was at position 248 (C → T), resulting in an amino acid change from serine to leucine at position 83^29^. Furthermore, this study used CRISPR/Cas9 to investigate the causal role of *gyr*A mutation in the quinolone resistance by inducing *gyr*A mutations in nucleotide 248 C to T and was able to successfully demonstrate the role of this mutation resulting in resistance to quinolone drugs.

CTX-M and *bla*-TEM are some of the predominant ESBLs, and are of specific relevance due to concerns of these genes in food-producing animals, including beef and dairy cattle^30–35^. A growing number of reports have indicated a timely apprehension with increased prevalence of ESBL *E. coli* isolates that are also resistant to quinolone drugs, given that cephalosporin and fluoroquinolone drugs are the common treatment of choice for infections caused by *E. coli* that require use of antimicrobial drugs^36–38^. Isolates carrying ESLB genes such as CTX-M have been frequently observed in isolates with resistance to multiple other antimicrobial drugs, and although specific factors resulting in this phenomena are not clear, co-selection is believed to play an essential role^39–42^. In our study, similar findings were observed with the two most prevalent MDR genotypic profiles characterized by simultaneously carrying resistance genes *bla*-TEM, *tet*A, and *tet*B (n = 73) and *aac(6′)Ib-cr,* CTX-M and *tet*A (n = 13), with 86% and 92% of these isolates, respectively, presenting phenotypic resistance to ciprofloxacin and ceftriaxone, in addition to other drugs (Table 4).

Plasmid-mediated quinolone resistance genes reduce susceptibility to fluoroquinolones and confer resistance to quinolones, which may help the selection of mutants with a high level of resistance^8^. Concerns around the spread of plasmids carrying resistance genes include the contribution to quinolone resistance, but also the potential risk for carrying resistance to β-lactams, aminoglycosides, tetracyclines or sulfonamides^43,44^. PMQR genes have been found on plasmids co-existing with ESBL genes, which can be transferred to recipient isolates^44,45^. In one study thirty-seven PMQR-harboring *E. coli* isolates were found in patients with bacteremia in a hospital in Taiwan, and among 10 of the isolates, β-lactamase resistance genes were also observed, namely *bla-*SHV-12, *bla* DHA-1, and *bla*CMY-2^46^. Fluoroquinolones, in addition to being a critical drug in human medicine, are also important and commonly used antimicrobial drugs to treat a wide range of bacterial infections in domestic animals. Liu et al. (2016) observed that PMQR genes were present in 80% of 40 cephalosporin-resistant *E. coli* isolates from dogs in China, and all were detected in co-existence with β-lactamase genes. Furthermore, an ESBL cattle surveillance program in England and Wales observed that one *E. coli* isolate contained the quinolone resistance gene *qnrS* while simultaneously carrying the β-lactamase resistance gene *blaTEM*, as well as other resistance genes conferring resistance to aminoglycoside, trimethoprim, sulphonamide, streptomycin and tetracycline drugs^47,48^.

For the two tetracycline genes screened in the study, *tet*A seems to persist at a higher prevalence at time points 0 and 7, with an overall gradual decrease observed at time points 28 and 56 (Table 3). A similar trend was observed for *tet*B, but with prevalence of *tet*B genes at timepoint 56 only observed in one isolate from a calf in the CTL group. Decrease overtime in prevalence of *tet*B resistance genes was also observed in another study with preweaned calves as they became older and closer to weaning age^49^. This shift in prevalence of tetracycline genes may be linked to the concept of a more mature microbiota beginning to install in the enteric environment, resulting in a more competitive scenario where fitness cost from carrying tetracycline genes may become overwhelming to bacteria.

In the current study, most breakpoints values used for susceptibility interpretation was based on the Clinical and Laboratory Standards Institute (CLSI) breakpoints for isolates of human origin. This is a common approach given the limited availability of interpretive criteria available to establish species-specific breakpoints for animals, the use of these reference values can pose as a pitfall for interpretation of isolates originating from cattle^50^. In recent years, more breakpoints have been generated to address this concern, and including veterinary interpretive criteria for veterinary drugs and species. However, current knowledge gaps still require the use of human based interpretive criteria for most isolates and drugs in veterinary settings.

## Conclusion

We observed that treatment of calves at high risk of developing BRD with enrofloxacin resulted in a significantly higher detection of mutation *gyr*A 248 at time points 7 and 28, and *aac(6′)Ib-cr* gene at time point 7 when compared the control group. These findings support a better understanding of the potential impacts from the use of enrofloxacin in preweaned calves on the selection and persistence of resistance caused by using this antimicrobial drug. Correlations between multidrug resistance genotypes and phenotypes observed also indicate the need for continued research to identify and understand factors corroborating for selection of multidrug resistant isolate that, if causing infections, can reduce the effectiveness of antimicrobial of critical importance. This is especially of relevance for those with simultaneous resistance to both fluoroquinolones and cephalosporin drugs.

## Material and methods

This study is part of a larger project on the effects of treatment with enrofloxacin or tulathromycin on antimicrobial resistant *E. coli* and fecal microbiota composition and function of preweaned dairy calves. The research protocol was reviewed and approved by the Institutional Animal Care and Use Committee of Cornell University (Protocol number: 2014-0094). All methods were performed in accordance with the relevant guidelines and regulations. Farm management and study design were described in Pereira et al. 2020^4^. Briefly, 84 calves not previously treated with antibiotics by the farm prior to study were enrolled between 2 to 3 weeks of age. Preweaned calves without clinical signs of pneumonia were randomly allocated to one of three study groups: (1) receiving a single subcutaneous dose of enrofloxacin (ENR) (7.5 mg/kg of body weight) in the neck following label directions (Baytril 100, Bayer Corp. Agricultural Division, Shawnee Mission, KS; 100 mg of enrofloxacin/mL); (2) receiving a single label dose of tulathromycin (TUL) (2.5 mg/kg of body weight) following label directions (Draxxin, Pfizer Animal Health, New York, NY; 100 mg of tulathromycin/mL); or (3) serving as a control and not receiving an antimicrobial drug treatment (CTL). Fecal samples from calves were collected longitudinally starting on the day of the administration of the antimicrobial treatment (day 0) and at 2, 4, 7, 14, 21, 28, 56, and 112 days after enrollment (9 time points total). Description of factors for defining and selection of these animals as high risk for BRD have been outlined in a previous publication^4^. Briefly, as defined in the U.S. Food and Drug Administration (FDA) new animal drug application (NADA) 141-068 and NADA 141-244, enrofloxacin and tulathromycin, are approved for use in beef and non-lactating dairy cattle for control of BRD in animals at high risk of developing BRD, and for treatment of BRD^51,52^. Through the NADA 141-068 the FDA defines the criteria that should be used to classify a population as being at high risk for BRD, and from the factors outlined in that document, the one that was identified on the farm used for this study where calves were sampled was the exposure of animals to wet or cold weather conditions.

### Isolation of resistant *Escherichia coli* and antimicrobial susceptibility testing using Kirby-Bauer disk diffusion

Fecal samples were processed cultures for *E. coli* and isolates tested for antimicrobial susceptibility, as previously described^20^. Briefly, diluted fecal samples were filtered through a hydrophobic grid membrane filter on MacConkey agar and replica-plated onto Mueller Hinton (MH) agar containing ciprofloxacin (1 µg/mL, ciprofloxacin hydrochloride; Alfa Aesar, Haverhill, MA), MH agar containing ceftriaxone (1 and 4 µg/mL, ceftriaxone sodium salt hemiheptahydrate; Acros Organics, Morris, New Jersey), MH with no antimicrobial drugs added, and chromogenic agar (CHROMagar *E. coli*; CHROMagar, Springfield, NJ) to confirm the identify *E. coli* colonies. A pitfall of using selective media is the potential for misidentification of isolate, whoever, previous research with CHROMagar *E. coli* has shown high accuracy for identifying *E. coli* isolates from fecal samples^53^. From those plates, one isolate that was, in order of preference and based on availability, resistant to both ciprofloxacin and ceftriaxone, resistant to only ciprofloxacin, resistant to only ceftriaxone was selected. If no isolate met one of these criteria, a susceptible isolate was selected. *E. coli* isolates were stored in Luria–Bertani broth containing 20% glycerol at − 80 °C.

Antibiotic susceptibility testing was conducted using a Kirby–Bauer disk diffusion agar assay in accordance with the guidelines published by the Clinical and Laboratory Standards Institute (CLSI) using a modified National Antimicrobial Resistance Monitoring System (NARMS) panel of 12 antimicrobial drugs: amoxicillin–clavulanic acid (Au), ampicillin (Am), cefoxitin (Fox), ceftriaxone (Cro), chloramphenicol (Cho), ciprofloxacin (Cip), nalidixic acid (Na), enrofloxacin (Enro), streptomycin (Str), sulfisoxazole (Sul), tetracycline (Te) and sulfamethoxazole-trimethoprim (Sxt) (Supplemental Table 1).

### Genomic DNA extraction

Two hundred sixty-four *E. coli* isolates that were phenotypically resistant to Ceftriaxone (CRO, n = 173) or to Ceftriaxone and Ciprofloxacin (CRO/CIP, n = 91), were selected for the genotypic characterization. One milliliter of grown culture was centrifuged at 12,000×g for 1 min and the pellet was frozen for later DNA extraction. Pellets were resuspended in 1 ml of nuclease free water (Life Technologies Corporation, Carlsbad, CA, USA), centrifuged and the supernatant removed. InstaGene matrix (Bio-Rad Laboratories, Inc. Hercules, CA, USA) was used following the manufacturer’s protocol. The DNA was stored at – 20 ℃.

### QRDR PCR and sequencing

Amplification of the quinolone resistance-determining regions (QRDR) of the *gyrA*, *parC*, *gyrB*, and *parE* genes was achieved using previously described PCR primers (Supplemental Table 2), which were checked against reference *E. coli* sequences and resistance genes in GenBank (NCBI) and on Primer3. The PCR was performed in 50 µl reactions consisting of 25 µl of Q5 Hot Start High-Fidelity 2X Master Mix (New England BioLabs Inc., Ipswich, MA, USA), 2.5 µl of each primer at 10 µM (reverse and forward), 18 µl nuclease free water and 2 µl DNA template. Thermo cycler conditions were an initial denaturing cycle at 98 °C for 30 s; followed by 30 cycles of 98 °C for 10 s, annealing temperature for 30 s, and 72 °C for 30 s; and a final extension step at 72 °C for 2 min. The annealing temperatures were 58 °C, 59 °C, 55 °C and 60 °C for *gyr*A, *gyr*B, *par*C and *par*E genes, respectively. Each of the 264 *E. coli* isolates was amplified for these 4 genes, resulting in 1,056 PCR reactions. The purified PCR products were submitted for Sanger sequencing at the Cornell University Institute of Biotechnology. The resulting sequences were uploaded to the Thermo Fisher Scientific online software for analysis using their Variant Analysis application (Thermo Fisher Scientific, Waltham, MA, USA).

### PMQR and *bla*-*tet* resistance genes multiplex PCR

Through multiplex PCR reactions, multiple genes may be amplified simultaneously. We used a touch-down method to decrease non-specific binding. Two multiplex PCR protocols were used, one for PMQR genes (qnrA, qnrD, qnrB, qnrS, oqxAB, Aac(6′)Ib-cr, qepA, and qnrC) and one for β-lactamase (bla-TEM, bla-CTX-M, bla-OXA) and tetracycline genes (tetA and tetB) (Supplemental Table 2). We focused screening to quinolone and β-lactam resistance genes based on a previous study that observed that farm records indicating prior treatment with enrofloxacin resulted in selection of multidrug resistant isolates in calves that presented phenotypic resistance to both ciprofloxacin and ceftriaxone^4^.

For the PMQR multiplex PCR, each 25 µl reaction had 12.5 µl of EconoTaq Green Master Mix (Lucigen, Middleton, WI, USA), 1 µl of DNA template, 8 µl of primer pool (8 primer pairs at 10 µM) and 3.5 µl of nuclease free water. The thermocycler conditions were an initial denaturing cycle at 94 °C for 5 min; followed by 16 cycles of 94 °C for 45 s, touchdown from 64.5 to 60 °C, decreasing 0.5 °C every 30 sec cycle, and 72 °C for 40 s; plus 15 cycles of 94 °C for 30 s, 60 °C for 90 s and 72 °C for 40 s, and a final extension step at 72 °C for 10 min.

The β-lactamase and tetracycline genes multiplex PCR protocol was developed in the laboratory. Each 25 µl reaction also had 12.5 µl of EconoTaq Green Master Mix and 1 µl of DNA template, however, 5 µl of primer pool were used and the volume of nuclease free water was increased to 6.5 µl. The cycling conditions were 94 °C for 5 min; 16 cycles of 94 °C for 45 s, touch-down from 66 °C to 58.5 °C, decreasing 0.5 °C every 30 s cycle, and 72 °C for 60 s; more 20 cycles of 94 °C for 45 s, 54 °C for 30 s and 72 °C for 60 s, and a final extension step at 72 °C for 10 min.

Every PCR plate had a positive control (pool of DNA from resistant *E.coli* and *Salmonella* isolates owned by our research lab) and a negative control (nuclease free water). PCR products were visualized in 2.0% agarose gels. Primers used on multiplex PCR reactions are also listed on Supplemental Table 2.

### Statistical analysis

Descriptive and chi-square analysis of data was conducted in JMP 15 (SAS Institute, Cary, NC, USA). This included a descriptive presentation of *E. coli* by point mutation according to phenotypic susceptibility classification for ciprofloxacin, as susceptible or intermediate (S/I) or resistant (R), and distribution of *E. coli* antimicrobial resistance gene profile and antimicrobial resistance phenotype profile. Heatmap of percent distribution by treatment group and time point for prevalence for antimicrobial resistance gene and QRDR mutations that were present in at least one isolate with phenotypic resistance to ciprofloxacin was created using RStudio version 1.2.5033.

Resistance gene prediction or QRDR mutation profile and corresponding expected phenotypic antimicrobial resistance was estimated using sensitivity (Se) and specificity (Sp). False negatives (FN) were defined as isolates not harboring the resistance gene or QRDR mutation profile but displaying expected phenotypic antimicrobial resistance, and true positive (TP) was defined as isolates harboring the resistance gene or QRDR mutation profile as well as displaying phenotypic resistance to the expected antimicrobial drug. Sensitivity was calculated using the formula Se = TP/TP + FN. False positives (FP) were defined as isolates harboring the resistance gene or QRDR mutation profile but not displaying expected phenotypic resistance, and true negative (TN) was defined as isolates not harboring the resistance gene or QRDR mutation profile and not displaying expected phenotypic resistance. Specificity was calculated using the formula Sp = TN/TN + FP. Isolates harboring the genes *aac(*6′)Ib-cr, CTX-M, *bla-*TEM, and *tet*A and *tet*B were expected to display phenotypic resistance to ciprofloxacin, ceftriaxone, ceftriaxone or ampicillin, and tetracycline, respectively. QRDR mutation profile was expected to display phenotypic resistance to ciprofloxacin.

To evaluate the association between resistance genes, point mutations at QRDR and QRDR point mutation profiles, and treatment groups for each time point, an initial screening was conducted using a Pearson chi-square test, with *P* value ≤ 0.10 used as a cut-off. Subsequently, resistance genes or point mutations at the selected QRDR, were offered to a mixed logistic regression model as the dependent variable, with treatment group and sampling time points as independent variables, including the interaction between these two variables (PROC GLIMMIX, SAS, SAS Institute, Cary, NC). Repeated measures were accounted for by offering individual animal identifiers sample identifier as a random effect in the model. Any association with a *P* value ≤ 0.05 was considered as significant. For resistance genes, point mutations at QRDR and QRDR point mutation that were not detected in any treatment groups for at least one time point, a two-sided Fisher exact test was used by time point using PROC FREQ in SAS to evaluate associations between treatment group and resistance genotype.

## Supplementary information

Supplementary Information.
