# Supplementary material for: Genotypic antimicrobial resistance characterization of E. coli from dairy calves at high risk of respiratory disease administered enrofloxacin or tulathromycin
Source: Sci Rep. 2020 Nov 9;10:19327. doi: 10.1038/s41598-020-76232-w (PMC7653923; doi:10.1038/s41598-020-76232-w)
Supplement: Supplementary file 1 — Supplementary Information. [file 41598_2020_76232_MOESM1_ESM.docx]

**Supplementary Material**

**Genotypic antimicrobial resistance characterization of *E. coli* from dairy calves at high risk of respiratory disease administered enrofloxacin or tulathromycin.**

R. V. Pereira^1^*, C. Foditsch^2^, J. D. Siler^2^, S. C. Dulièpre^2^, C. Altier^2^, A. Garzon^1^, L. D. Warnick^2^.

1. Department of Population Health and Reproduction, College of Veterinary Medicine, University of California Davis, Davis, CA, 95616; 2. Department of Population Medicine and Diagnostic Sciences, College of Veterinary Medicine, Cornell, University, Ithaca, NY, 14850.

*RVPereira@UCDavis.edu

**Supplemental Table 1.** Zone diameter breakpoints used for isolates in the study.

|  | Zone Diameter (mm) ^1^ | | |
| --- | --- | --- | --- |
| Antimicrobial Drug | **S**^2^ | **I**^3^ | **R**^4^ |
| Amoxicillin Clavulanic Acid | ≥ 18 | 14 - 17 | ≤ 13 |
| Ampicillin | ≥ 17 | 14 - 16 | ≤ 13 |
| Cefoxitin | ≥ 18 | 15 - 17 | ≤ 14 |
| Ceftriaxone | ≥ 23 | 20 - 22 | ≤ 19 |
| Chloramphenicol | ≥ 18 | 13 - 17 | ≤ 12 |
| Ciprofloxacin | ≥ 21 | 16 - 20 | ≤ 15 |
| Enrofloxacin | ≥ 22 | 17 - 21 | ≤ 16 |
| Nalidixic Acid | ≥ 19 | 14 - 18 | ≤ 13 |
| Streptomycin | ≥ 15 | 12 - 14 | ≤ 11 |
| Sulfamethoxazole- Trimethoprim | ≥ 16 | 11 - 15 | ≤ 10 |
| Sulfisoxazole | ≥ 17 | 13 - 16 | ≤ 12 |
| Tetracycline | ≥ 15 | 12 - 14 | ≤ 11 |

1. Source of breakpoint values: CLSI M100-S22 Vol.32 No.3. and CLSI VET01-S2.
2. Susceptible to referred antimicrobial.
3. Intermediate to referred antimicrobial.
4. Resistant to referred antimicrobial.

**Supplemental Table 2.** Primers descriptive information and references.

|  | **Primer** | | **Sequence (5'- 3')** | **Tm (°C)** | | **Fragment**  **size (bp)** | | **Ref.** |
| --- | --- | --- | --- | --- | --- | --- | --- | --- |
| **QRDR** | gyrA | F | TACACCGGTCAACATTGAGG | | 55 | | 648 | *^1^* |
|  |  | R | TTAATGATTGCCGCCGTCGG | | 58 | |  |  |
|  | gyrB | F | CTCCTCCCAGACCAAAGACA | | 56 | | 461 | *^2^* |
|  |  | R | TCACGACCGATACCACAGCC | | 59 | |  |  |
|  | parC | F | TGTATGCGATGTCTGAACTG | | 52 | | 265 | *^3^* |
|  |  | R | CTCAATAGCAGCTCGGAATA | | 52 | |  |  |
|  | parE | F | TACCGAGCTGTTCCTTGTGG | | 57 | | 265 | *^3^* |
|  |  | R | GGCAATGTGCAGACCATCAG | | 57 | |  |  |
| **PMQR multiplex** | qnrA | F | CAGCAAGAGGATTTCTCACG | | 53 | | 631 | *^4^* |
|  |  | R | AATCCGGCAGCACTATTACTC | | 55 | |  |  |
|  | qnrD | F | CGAGATCAATTTACGGGGAATA | | 52 | | 582 | *^4,5^* |
|  |  | R | AACAAGCTGAAGCGCCTG | | 56 | |  |  |
|  | qnrB | F | GGCTGTCAGTTCTATGATCG | | 53 | | 489 | *^4^* |
|  |  | R | GAGCAACGACGCCTGGTAG | | 58 | |  |  |
|  | qnrS | F | GCAAGTTCATTGAACAGGGT | | 53 | | 427 | *^4,6^* |
|  |  | R | TCTAAACCGTCGAGTTCGGCG | | 59 | |  |  |
|  | oqxAB | F | CCGCACCGATAAATTAGTCC | | 53 | | 313 | *^4^* |
|  |  | R | GGCGAGGTTTTGATAGTGGA | | 55 | |  |  |
|  | Aac(6')Ib-cr | F | TTGGAAGCGGGGACGGAC | | 60 | | 264 | *^4,7^* |
|  |  | R | TACACGGCTGGACCATAT | | 53 | |  |  |
|  | qepA | F | GCAGGTCCAGCAGCGGGTAG | | 63 | | 218 | *^8^* |
|  |  | R | CTTCCTGCCCGAGTATCGTG | | 58 | |  |  |
|  | qnrC | F | GCAGAATTCAGGGGTGTGAT | | 55 | | 118 | *^4^* |
|  |  | R | AACTGCTCCAAAAGCTGCTC | | 56 | |  |  |
| **Bla-tet multiplex** | blaTEM | F | CGCCGCATACACTATTCTCAGAATGA | | 63 | | 444 | *^9^* |
|  |  | R | ACGCTCACCGGCTCCAGATTTAT | | 64 | |  |  |
|  | blaCTX-M | F | ATGTGCAGYACCAGTAARGTKATGGC | | 66 | | 593 | *^9-11^* |
|  |  | R | TGGGTRAARTARGTSACCAGAAYCAGCGG | | 68 | |  |  |
|  | blaOXA | F | ACACAATACATATCAACTTCGC | | 55 | | 814 | *^9^* |
|  |  | R | AGTGTGTTTAGAATGGTGATC | | 54 | |  |  |
|  | tet(A) | F | GCTACATCCTGCTTGCCTTC | | 59 | | 210 | *^12,13^* |
|  |  | R | CATAGATCGCCGTGAAGAGG | | 58 | |  |  |
|  | tet(B) | F | TTGGTTAGGGGCAAGTTTTG | | 56 | | 659 | *^12,13^* |
|  |  | R | GTAATGGGCCAATAACACCG | | 57 | |  |  |

**REFERENCES**

1 Oram, M. & Fisher, L. M. 4-Quinolone resistance mutations in the DNA gyrase of Escherichia coli clinical isolates identified by using the polymerase chain reaction. *Antimicrob Agents Chemother* **35**, 387-389, doi:10.1128/aac.35.2.387 (1991).

2 Vila, J. *et al.* Association between double mutation in gyrA gene of ciprofloxacin-resistant clinical isolates of *Escherichia coli* and MICs. *Antimicrob Agents Chemother* **38**, 2477-2479, doi:10.1128/aac.38.10.2477 (1994).

3 Everett, M. J., Jin, Y. F., Ricci, V. & Piddock, L. J. Contributions of individual mechanisms to fluoroquinolone resistance in 36 *Escherichia coli* strains isolated from humans and animals. *Antimicrob Agents Chemother* **40**, 2380-2386 (1996).

4 Ciesielczuk, H., Hornsey, M., Choi, V., Woodford, N. & Wareham, D. W. Development and evaluation of a multiplex PCR for eight plasmid-mediated quinolone-resistance determinants. *Journal of medical microbiology* **62**, 1823-1827, doi:10.1099/jmm.0.064428-0 [doi] (2013).

5 Cavaco, L. M., Hasman, H., Xia, S. & Aarestrup, F. M. qnrD, a novel gene conferring transferable quinolone resistance in *Salmonella* *enterica* serovar Kentucky and Bovismorbificans strains of human origin. *Antimicrob Agents Chemother* **53**, 603-608, doi:10.1128/AAC.00997-08 (2009).

6 Cattoir, V., Poirel, L., Rotimi, V., Soussy, C. J. & Nordmann, P. Multiplex PCR for detection of plasmid-mediated quinolone resistance qnr genes in ESBL-producing enterobacterial isolates. *J Antimicrob Chemother* **60**, 394-397, doi:10.1093/jac/dkm204 (2007).

7 Wareham, D. W., Umoren, I., Khanna, P. & Gordon, N. C. Allele-specific polymerase chain reaction (PCR) for rapid detection of the *aac(6')-Ib-cr* quinolone resistance gene. *Int J Antimicrob Agents* **36**, 476-477, doi:10.1016/j.ijantimicag.2010.07.012 (2010).

8 Yamane, K., Wachino, J., Suzuki, S. & Arakawa, Y. Plasmid-mediated qepA gene among *Escherichia coli* clinical isolates from Japan. *Antimicrob Agents Chemother* **52**, 1564-1566, doi:10.1128/AAC.01137-07 (2008).

9 Fang, H., Ataker, F., Hedin, G. & Dornbusch, K. Molecular epidemiology of extended-spectrum beta-lactamases among *Escherichia coli* isolates collected in a Swedish hospital and its associated health care facilities from 2001 to 2006. *J Clin Microbiol* **46**, 707-712, doi:10.1128/JCM.01943-07 (2008).

10 Monstein, H. J. *et al.* Multiplex PCR amplification assay for the detection of *bla*SHV, *bla*TEM and *bla*CTX-M genes in Enterobacteriaceae. *APMIS* **115**, 1400-1408, doi:10.1111/j.1600-0463.2007.00722.x (2007).

11 Moubareck, C. *et al.* Countrywide spread of community- and hospital-acquired extended-spectrum beta-lactamase (CTX-M-15)-producing Enterobacteriaceae in Lebanon. *J Clin Microbiol* **43**, 3309-3313, doi:10.1128/JCM.43.7.3309-3313.2005 (2005).

12 Fan, W., Hamilton, T., Webster-Sesay, S., Nikolich, M. P. & Lindler, L. E. Multiplex real-time SYBR Green I PCR assay for detection of tetracycline efflux genes of Gram-negative bacteria. *Mol Cell Probes* **21**, 245-256, doi:10.1016/j.mcp.2006.12.005 (2007).

13 Ng, L. K., Martin, I., Alfa, M. & Mulvey, M. Multiplex PCR for the detection of tetracycline resistant genes. *Mol Cell Probes* **15**, 209-215, doi:10.1006/mcpr.2001.0363 (2001).

**Supplemental Table 3**. Association of *E coli* by quinolone resistant-determining regions point mutation according to phenotypic susceptibility classification for ciprofloxacin, as susceptible or intermediate (S/I) and resistant (R).

| Point Mutation | Ciprofloxacin, % (n) | | | |
| --- | --- | --- | --- | --- |
|  | **S/I (n=186)** ^1^ | **R (n=78)** ^2^ | **Chi-Square**^3^ | ***P* value**^4^ |
| *gyr*A 594: T → C | 90 (167) | 99 (77) | 0.42 | 0.51 |
| *par*C 239: G → T | 1 (2) | 99 (77) | 249.8 | **<.0001** |
| *gyr*A 333: T → C | 90 (168) | 96 (75) | 2.52 | 0.11 |
| *gyr*A 273: C → T | 90 (168) | 96 (75) | 2.55 | 0.11 |
| *gyr*A 255: C → T | 90 (168) | 96 (75) | 2.55 | 0.11 |
| *gyr*A 300: T → C | 89 (166) | 96 (75) | 3.30 | 0.07 |
| *gyr*A 248: C → T | 2 (3) | 95 (74) | 231.3 | **<.0001** |
| *gyr*A 259: G → A | 1 (2) | 95 (74) | 235.8 | **<.0001** |
| *gyr*A 570: C → T | 3 (5) | 78 (61) | 167.1 | **<.0001** |
| *par*E 1372: T → G | 1 (1) | 78 (61) | 184.5 | **<.0001** |
| *par*C 273: A → G | 85 (159) | 0 (0) |  |  |
| *par*E 1386: C → T | 57 (106) | 0 (0) |  |  |
| *par*E 1371: T → C | 57 (106) | 0 (0) |  |  |
| *gyr*B 1152: G → T | 26 (49) | 0 (0) |  |  |
| *gyr*B 1359: C → T | 24 (45) | 0 (0) |  |  |
| *gyr*B 1266: G → T | 20 (38) | 0 (0) |  |  |
| *par*E 1446: T → C | 19 (36) | 0 (0) |  |  |
| *par*C 348: G → A | 17 (32) | 0 (0) |  |  |
| *gyr*B 1410: T → G | 12 (22) | 0 (0) |  |  |
| *gyr*B 1377: T → C | 12 (22) | 0 (0) |  |  |
| *gyr*B 1305: G → A | 11 (20) | 0 (0) |  |  |
| *par*C 192: C → T | 11 (20) | 0 (0) |  |  |
| *par*E 1380: A → G | 10 (19) | 0 (0) |  |  |
| *par*E 1353: T → C | 10 (19) | 0 (0) |  |  |
| *gyr*B 1161: C → T | 10 (18) | 0 (0) |  |  |
| *gyr*B 1125: T → C | 10 (18) | 0 (0) |  |  |
| *par*E 1308: G → T | 9 (17) | 0 (0) |  |  |
| *gyr*B 1284: G → A | 9 (17) | 0 (0) |  |  |
| *gyr*B 1392: T → C | 9 (16) | 0 (0) |  |  |
| *gyr*B 1116: T → C | 9 (16) | 0 (0) |  |  |
| *gyr*B 1113: G → A | 9 (16) | 0 (0) |  |  |
| *par*C 240: C → T | 9 (16) | 0 (0) |  |  |
| *gyr*A 105: A → G | 9 (16) | 0 (0) |  |  |
| *gyr*B 1254: C → T | 8 (15) | 0 (0) |  |  |
| *gyr*B 1251: T → C | 8 (15) | 0 (0) |  |  |
| *gyr*B 1197: C → T | 8 (15) | 0 (0) |  |  |
| *gyr*B 1194: C → G | 8 (15) | 0 (0) |  |  |
| *gyr*B 1179: C → T | 8 (14) | 0 (0) |  |  |
| *gyr*B 1137: C → T | 8 (14) | 0 (0) |  |  |
| *gyr*A 480: A → G | 8 (14) | 0 (0) |  |  |
| *gyr*B 1242: T → C | 7 (13) | 0 (0) |  |  |
| *gyr*B 1230: C → T | 7 (13) | 0 (0) |  |  |
| *gyr*B 1215: C → T | 7 (13) | 0 (0) |  |  |
| *gyr*B 1074: A → G | 7 (13) | 0 (0) |  |  |
| *gyr*A 552: G → A | 7 (13) | 0 (0) |  |  |
| *par*C 270: G → A | 7 (13) | 0 (0) |  |  |
| *par*C 243: C → T | 6 (12) | 0 (0) |  |  |
| *gyr*B 1212: G → A | 6 (11) | 0 (0) |  |  |
| *gyr*B 1224: A → C | 5 (9) | 0 (0) |  |  |
| *gyr*A 438: T → A | 5 (9) | 0 (0) |  |  |
| *gyr*A 639: G → C | 4 (8) | 0 (0) |  |  |
| *gyr*B 1164: G → A | 4 (8) | 0 (0) |  |  |
| *par*E 1429: C → T | 4 (8) | 0 (0) |  |  |
| *par*E 1401: G → T | 4 (8) | 0 (0) |  |  |
| *par*E 1473: G → T | 3 (6) | 0 (0) |  |  |
| *par*E 1464: C → G | 3 (6) | 0 (0) |  |  |
| *par*E 1452: A → G | 3 (6) | 0 (0) |  |  |
| *par*E 1317: A → G | 3 (5) | 0 (0) |  |  |
| *par*C 321: C → T | 2 (4) | 0 (0) |  |  |
| *par*E 1296: C → T | 2 (4) | 0 (0) |  |  |
| *gyr*A 513: T → C | 2 (4) | 0 (0) |  |  |
| *gyr*A 126: G → A | 2 (4) | 0 (0) |  |  |
| *par*E 1350: C → A | 2 (3) | 0 (0) |  |  |
| *gyr*B 1101: C → T | 2 (3) | 0 (0) |  |  |
| *gyr*B 1098: C → T | 2 (3) | 0 (0) |  |  |
| *gyr*A 468: G → T | 1 (2) | 0 (0) |  |  |
| *par*E 1305: G → A | 1 (2) | 0 (0) |  |  |
| *par*E 1428: T → C | 1 (2) | 0 (0) |  |  |
| *gyr*B 1365: A → G | 1 (2) | 0 (0) |  |  |
| *gyr*B 1353: C → T | 1 (2) | 0 (0) |  |  |
| *gyr*B 1341: G → A | 1 (2) | 0 (0) |  |  |
| *par*E 1332: C → T | 1 (2) | 0 (0) |  |  |
| *gyr*B 1329: T → C | 1 (2) | 0 (0) |  |  |
| *gyr*B 1245: G → C | 1 (2) | 0 (0) |  |  |
| *gyr*B 1236: A → G | 1 (2) | 0 (0) |  |  |
| *gyr*B 1203: G → T | 1 (2) | 0 (0) |  |  |
| *gyr*B 1104: G → A | 1 (2) | 0 (0) |  |  |
| *gyr*B 1068: G → C | 1 (2) | 0 (0) |  |  |
| *gyr*A 408: C → T | 1 (2) | 0 (0) |  |  |
| *par*C 375: G → A | 1 (2) | 0 (0) |  |  |
| *par*C 351: A → G | 1 (2) | 0 (0) |  |  |
| *par*C 324: G → A | 1 (2) | 0 (0) |  |  |
| *par*C 295: C → T | 1 (2) | 0 (0) |  |  |
| *gyr*A 252: G → A | 1 (2) | 0 (0) |  |  |
| *par*C 234: C → T | 1 (2) | 0 (0) |  |  |
| *par*E 1479: T → C | 1 (1) | 0 (0) |  |  |
| *par*E 1467: G → A | 1 (1) | 0 (0) |  |  |
| *par*E 1437: G → A | 1 (1) | 0 (0) |  |  |
| *par*E 1425: C → T | 1 (1) | 0 (0) |  |  |
| *par*E 1416: T → A | 1 (1) | 0 (0) |  |  |
| *gyr*B 1308: T → C | 1 (1) | 0 (0) |  |  |
| *gyr*B 1278: C → T | 1 (1) | 0 (0) |  |  |
| *gyr*B 1258: C → T | 1 (1) | 0 (0) |  |  |
| *gyr*B 1207: C → T | 1 (1) | 0 (0) |  |  |
| *gyr*A 642: G → T | 1 (1) | 0 (0) |  |  |
| *gyr*A 576: C → T | 1 (1) | 0 (0) |  |  |
| *gyr*A 537: A → G | 1 (1) | 0 (0) |  |  |
| *gyr*A 453: C → T | 1 (1) | 0 (0) |  |  |
| *gyr*A 315: T → C | 1 (1) | 0 (0) |  |  |
| *gyr*A 99: G → A | 1 (1) | 0 (0) |  |  |

1. Percent distribution of isolate with the identified mutation within the population of isolates classified as susceptible or intermediate to ciprofloxacin. Count number in *par*enthesis
2. Percent distribution of isolate with the identified mutation within the population of isolates classified as resistant to ciprofloxacin. Count number in *par*enthesis.
3. Pearson Chi-square test for the association between the referred chromosomal point mutation and phenotypic resistance to ciprofloxacin.
4. *P* value for Pearson Chi-square test.

**Supplemental Table 4.** Distribution of the accumulated QRDR mutations observed in individual isolates (QRDR profiles)

| QRDR Mutation Profile | S/I (n=186)^1^ | R (n=78)^2^ | Total^3^ |
| --- | --- | --- | --- |
| *gyrA_468 gyrA_273 gyrA_255 gyrA_333 gyrA_300 parC_273 parE_1386 parE_1350 parE_1371* | 24 (60) | 0 (0) | 24 (60) |
| *parC_239 gyrA_248 gyrA_259 gyrA_570 parE_1372 gyrA_468 gyrA_273 gyrA_255 gyrA_333 gyrA_300* | 24 (1) | 98 (58) | 24 (59) |
| *gyrA_468 gyrA_273 gyrA_255 gyrA_333 gyrA_300 parC_273* | 13 (33) | 0 (0) | 13 (33) |
| *gyrA_468 gyrA_273 gyrA_255 gyrA_333 gyrA_300 gyrB_1359 parC_273 parE_1386 parE_1350 parE_1371* | 12 (30) | 0 (0) | 12 (30) |
| *parC_239 gyrA_248 gyrA_259 gyrA_468 gyrA_273 gyrA_255 gyrA_333 gyrA_300* | 6 (1) | 93 (14) | 6 (15) |
| *gyrA_273 gyrA_255 gyrA_333 gyrA_300 gyrB_1164 parC_273* | 5 (13) | 0 (0) | 5 (13) |
| gyrA_468 gyrA_273 gyrA_255 gyrA_333 gyrA_300 | 3 (7) | 0 (0) | 3 (7) |
| *gyrA_468 gyrA_273 gyrA_255 gyrA_333 gyrA_300 gyrB_1359 parC_273 parE_1386 parE_1371* | 3 (7) | 0 (0) | 3 (7) |
| *gyrA_570 gyrA_273 gyrA_255 gyrA_333 gyrA_300 parC_273* | 2 (4) | 0 (0) | 2 (4) |
| *gyrA_273 gyrA_255 gyrA_333 gyrA_300 parC_273* | 1 (2) | 0 (0) | 1 (2) |
| *gyrA_468 gyrA_273 gyrA_255 gyrA_333 gyrA_300 parE_1386 parE_1350 parE_1371* | 1 (2) | 0 (0) | 1 (2) |
| *gyrA_468 gyrA_273 gyrA_255 gyrA_333 gyrB_1359 parC_273 parE_1386 parE_1350 parE_1371* | 1 (2) | 0 (0) | 1 (2) |
| *parC_239 gyrA_248 gyrA_259 gyrA_570 gyrA_468 gyrA_273 gyrA_255 gyrA_333 gyrA_300* | 1 (0) | 100 (2) | 1 (2) |
| *gyrA_248 gyrA_468 gyrA_273 gyrA_255 gyrA_333 gyrA_300 gyrB_1359 parC_273 parE_1386 parE_1350 parE_1371* | 0 (1) | 0 (0) | 0 (1) |
| *gyrA_273 gyrA_255 gyrA_333 gyrA_300 gyrB_1359 parC_273* | 0 (1) | 0 (0) | 0 (1) |
| *gyrA_273 gyrA_255 gyrA_333 gyrA_300 gyrB_1359 parC_273 parE_1386 parE_1350 parE_1371* | 0 (1) | 0 (0) | 0 (1) |
| *gyrA_468 gyrA_273 gyrA_255 gyrA_333 gyrA_300 gyrB_1164 parC_273* | 0 (1) | 0 (0) | 0 (1) |
| *gyrA_468 gyrA_273 gyrA_255 gyrA_333 gyrA_300 gyrB_1359 parC_273* | 0 (1) | 0 (0) | 0 (1) |
| *gyrA_468 gyrA_594 gyrA_273 gyrA_255 gyrA_333 gyrA_300 parC_273 parE_1386 parE_1350 parE_1371* | 0 (1) | 0 (0) | 0 (1) |
| *gyrB_1359 parC_273 parE_1386 parE_1350 parE_1371* | 0 (1) | 0 (0) | 0 (1) |
| *gyrB_1359 parC_273 parE_1386 parE_1371* | 0 (1) | 0 (0) | 0 (1) |
| *parC_239 gyrA_570 parE_1372 gyrA_468* | 0 (0) | 100 (1) | 0 (1) |
| *parC_239 parE_1372 gyrA_468* | 0 (0) | 100 (1) | 0 (1) |
| *parC_239 parE_1372 gyrA_468 gyrA_273 gyrA_255 gyrA_333 gyrA_300* | 0 (0) | 100 (1) | 0 (1) |

1. Percent (count in parenthesis) distribution of isolate with the identified QRDR mutation profile within the population of isolates classified as susceptible or intermediate to ciprofloxacin.

2. Percent (count in parenthesis) distribution of isolate with the identified QRDR mutation within the population of isolates classified as resistant to ciprofloxacin.

3. Percent (count in parenthesis) of isolates with the QRDR mutation profile.
